# Supplementary material for: Point-of-care infrared thermal imaging for differentiating venomous snakebites from non-venomous and dry bites
Source: PLoS Negl Trop Dis. 2021 Feb 18;15(2):e0008580. doi: 10.1371/journal.pntd.0008580 (PMC7924804; doi:10.1371/journal.pntd.0008580)

# 1a. Envenomation+ Hot spot+

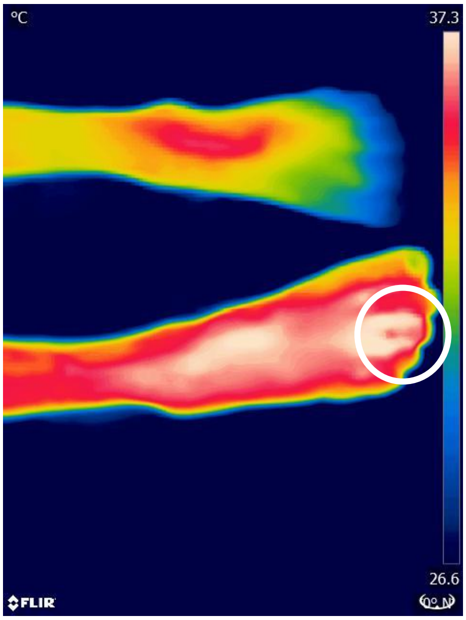


#4

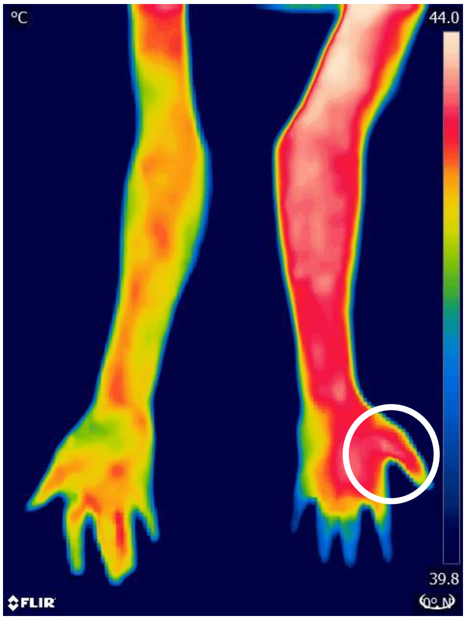


#7

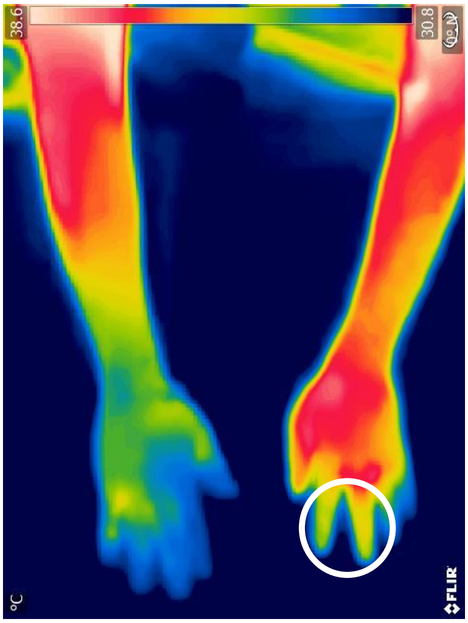


#9

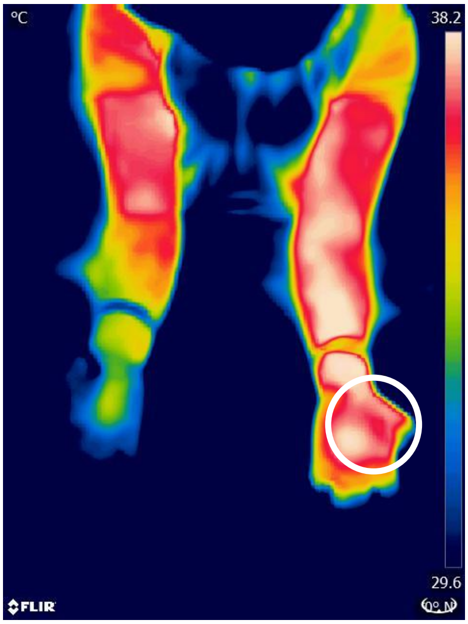


#10

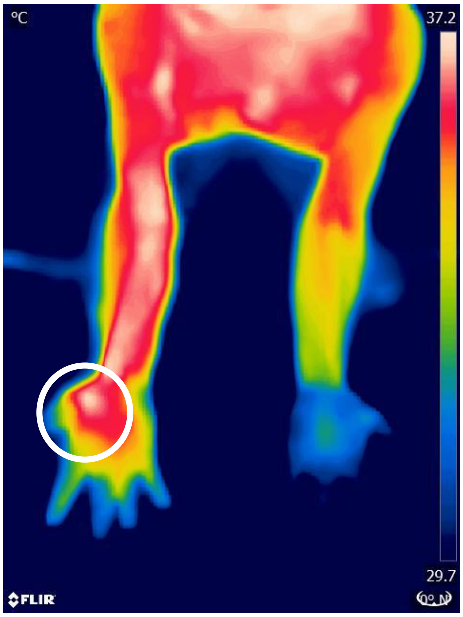


#13

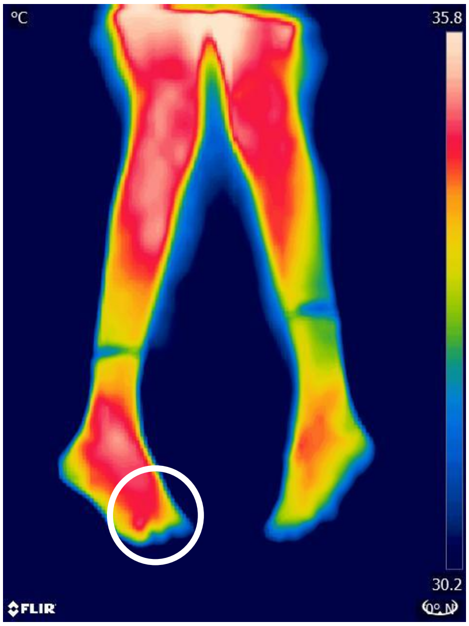


#18

# 1a. Envenomation+ Hot spot+

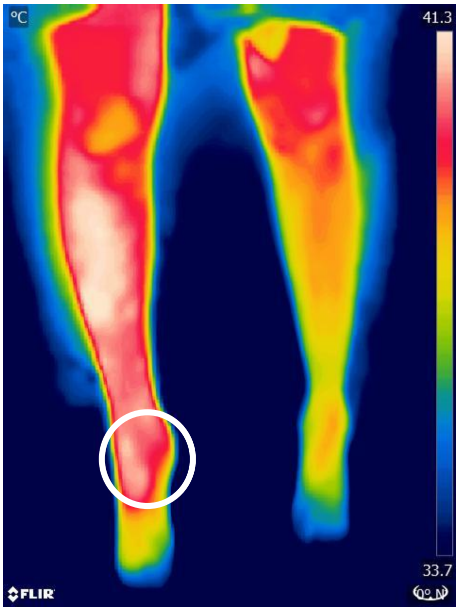


#20

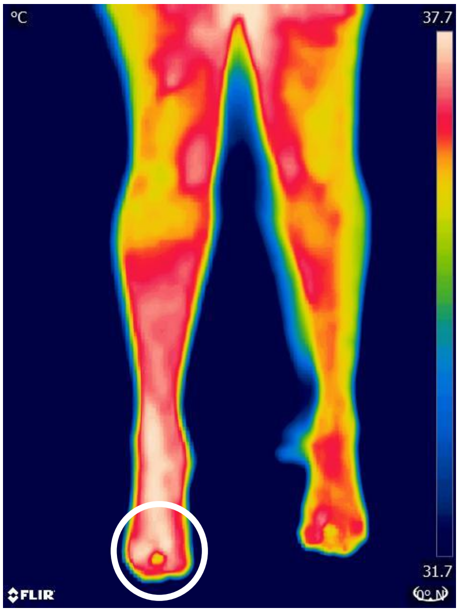


#22

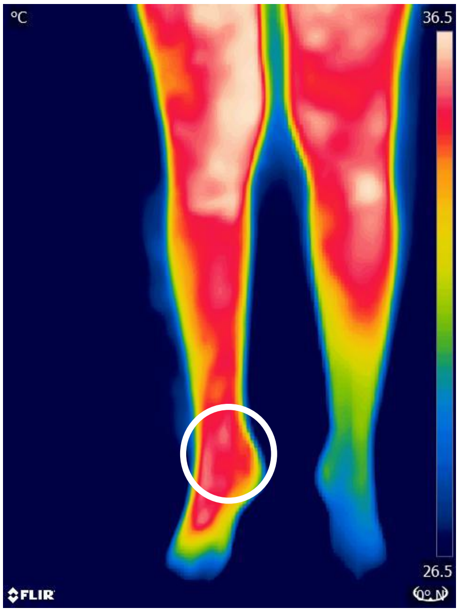


#24

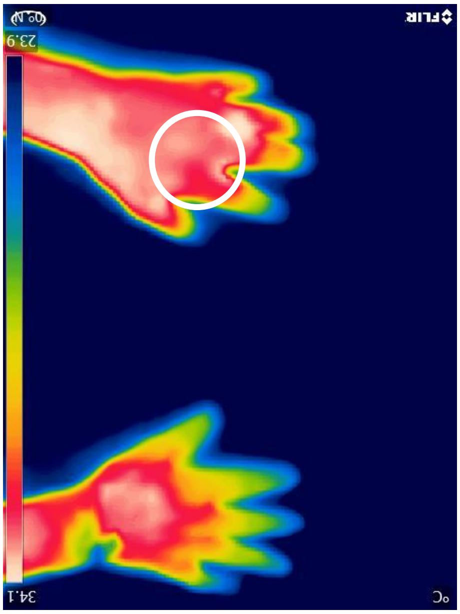


#28

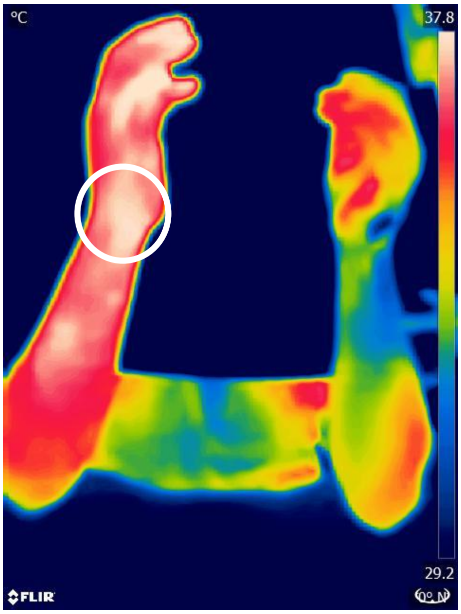


#30

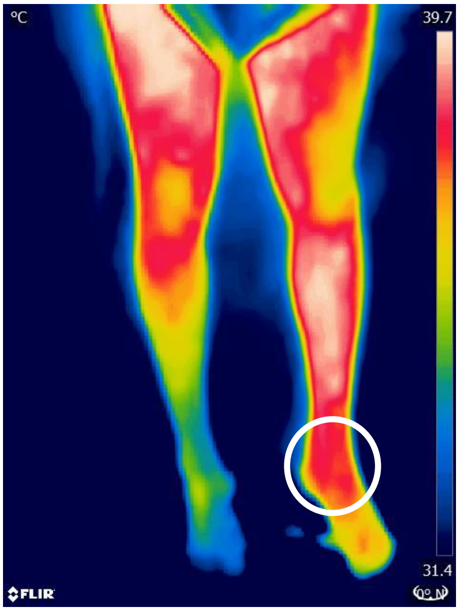


#32

# 1a. Envenomation+ Hot spot+

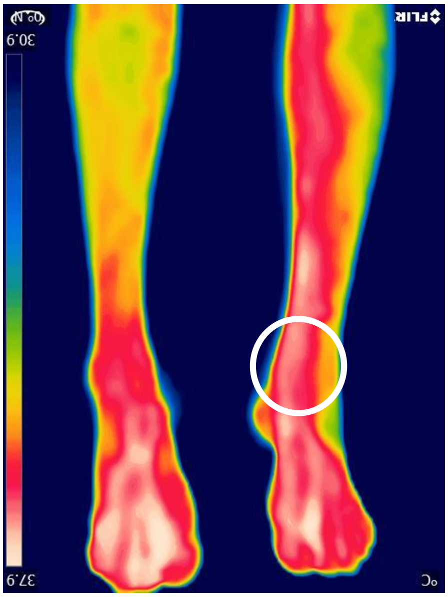

#35

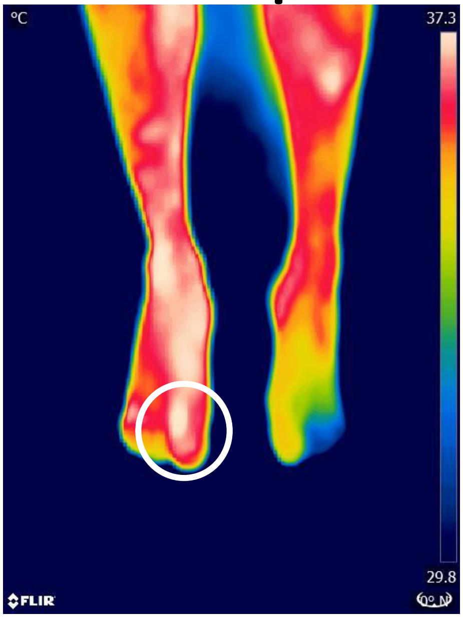


#36

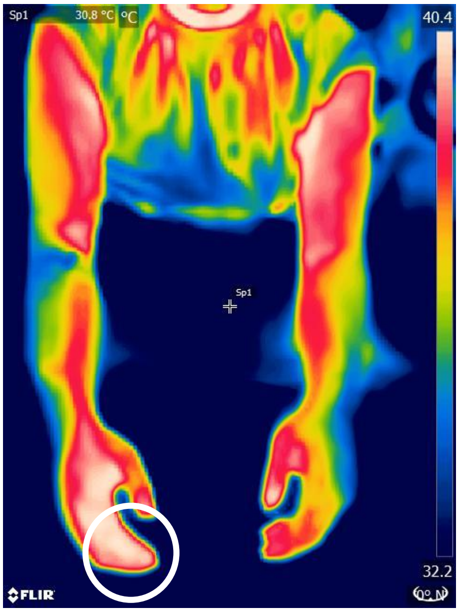


#39

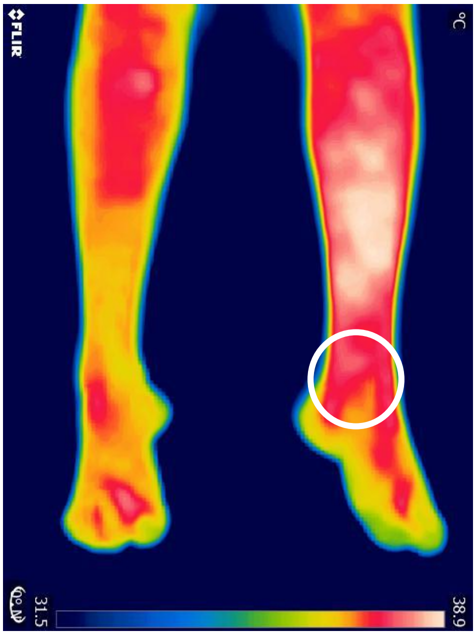

#41

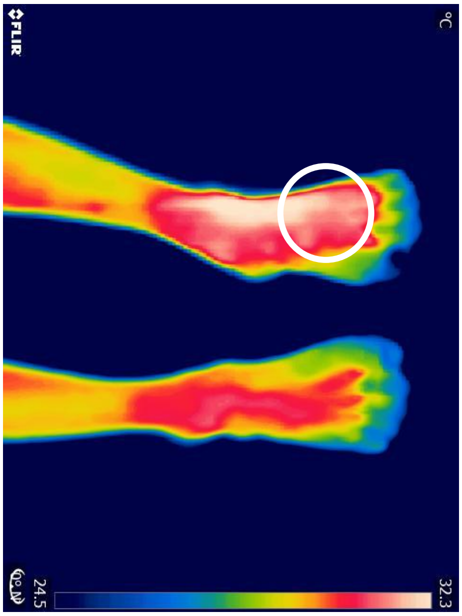

#42

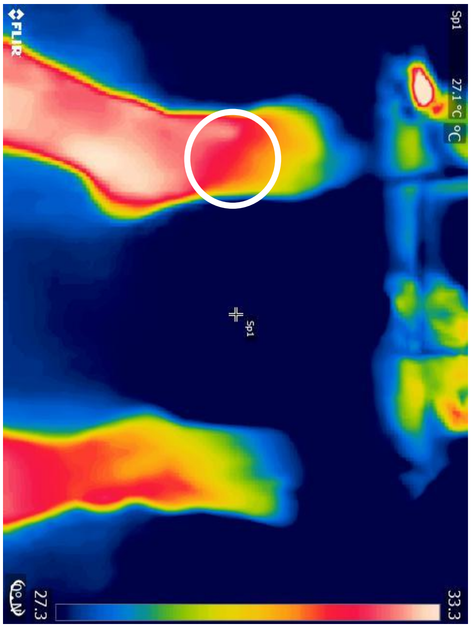

#46

# 1a. Envenomation+ Hot spot+

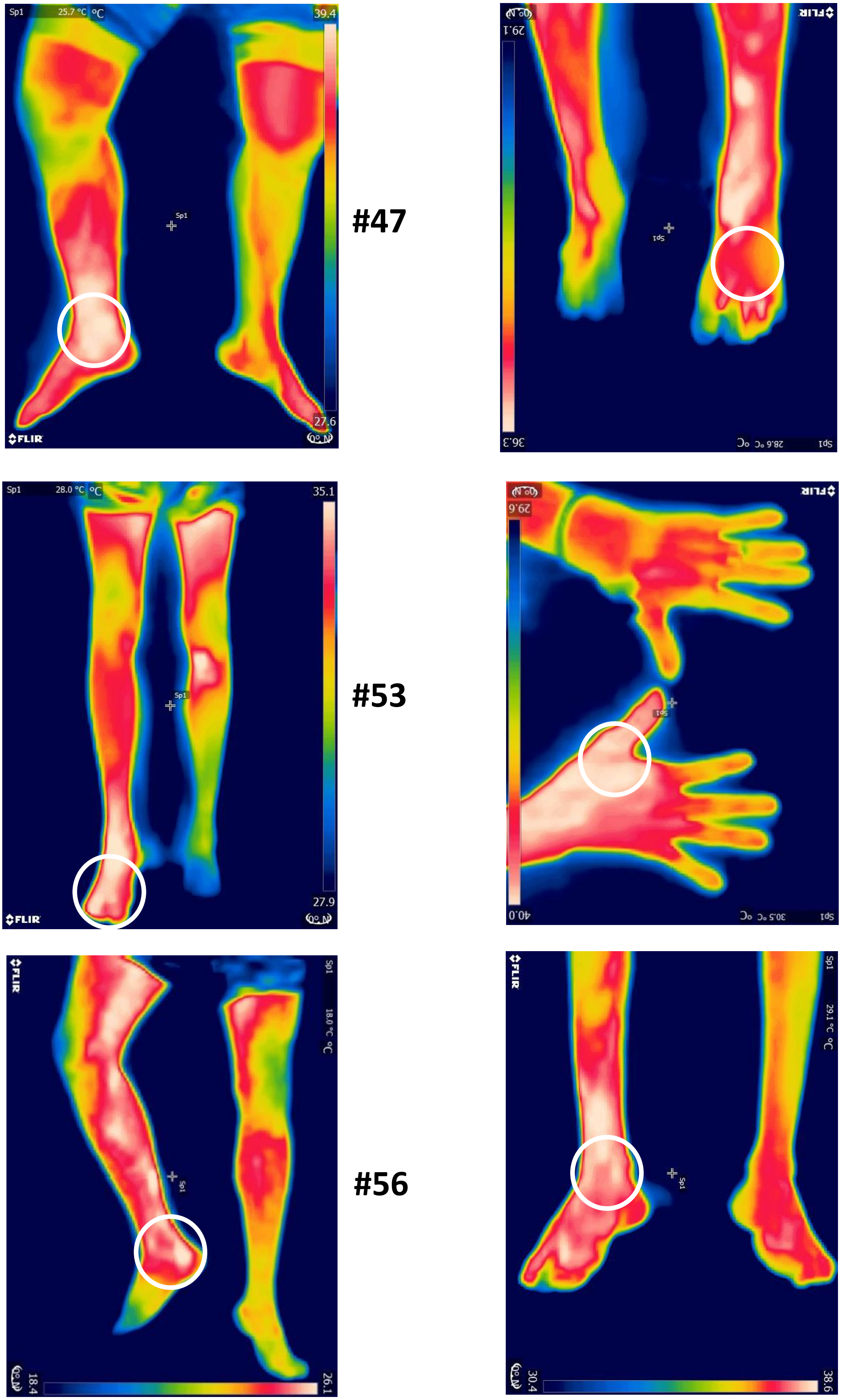


#47

#50

#53

#54

#56

#58

# 1a. Envenomation+ Hot spot+

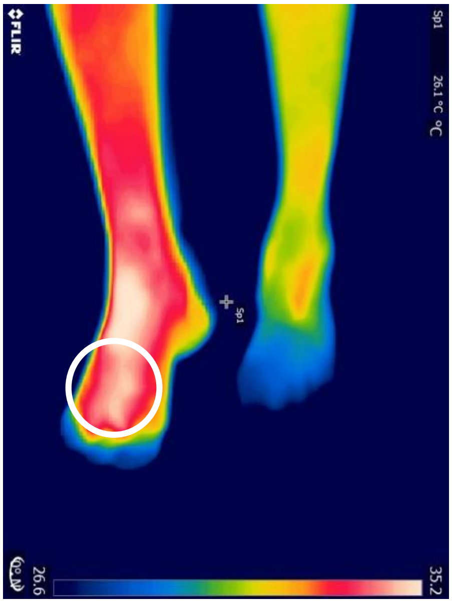


#60

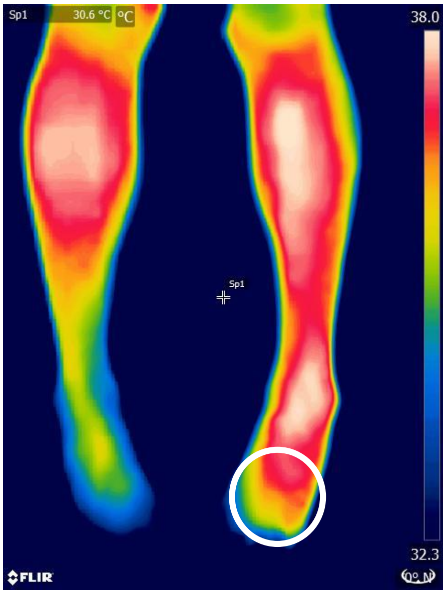


#61

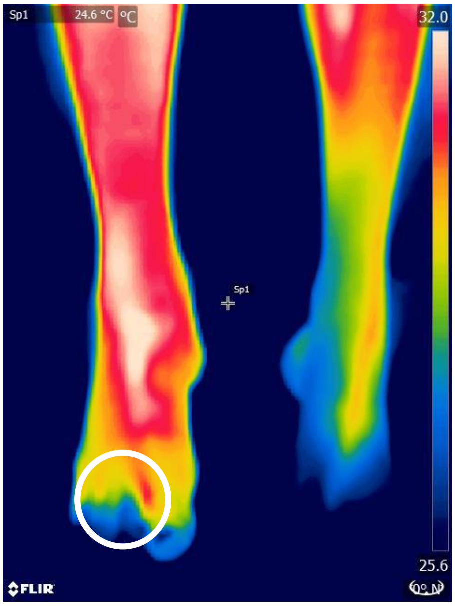


#64

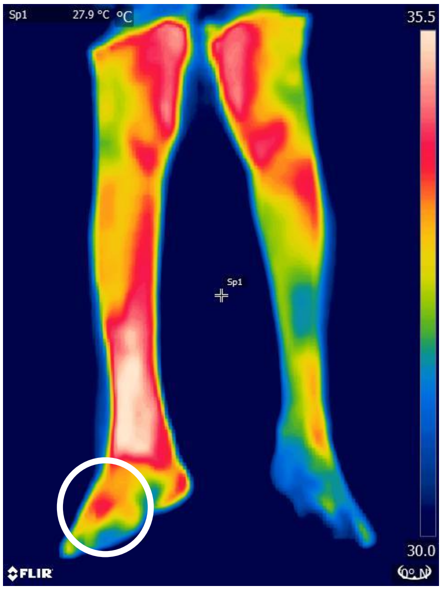


#65

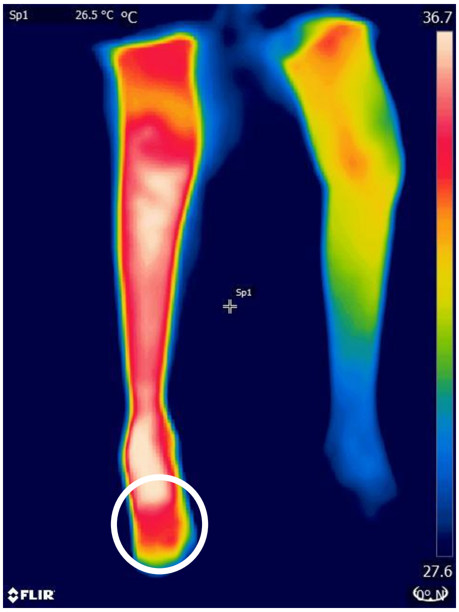


#66

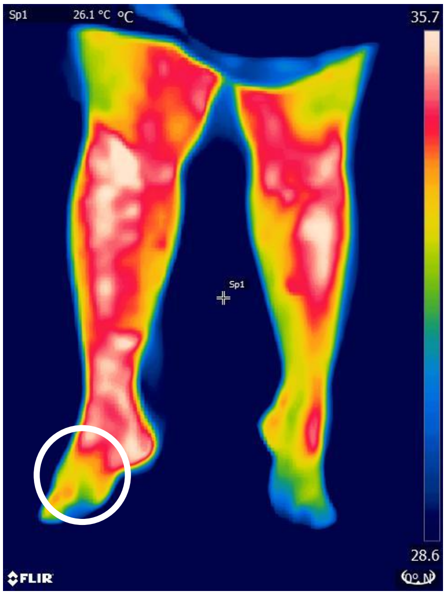


#67

# 1a. Envenomation+ Hot spot+

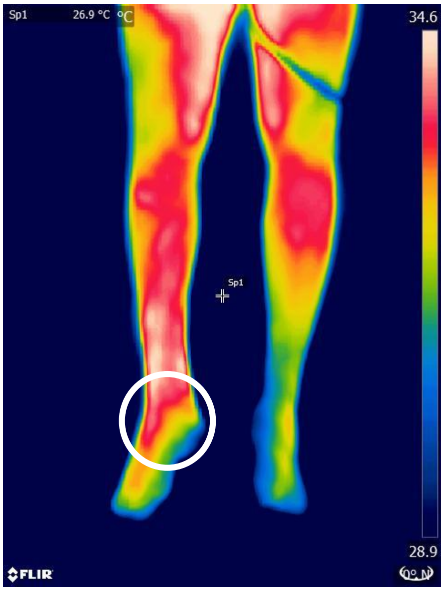


#68

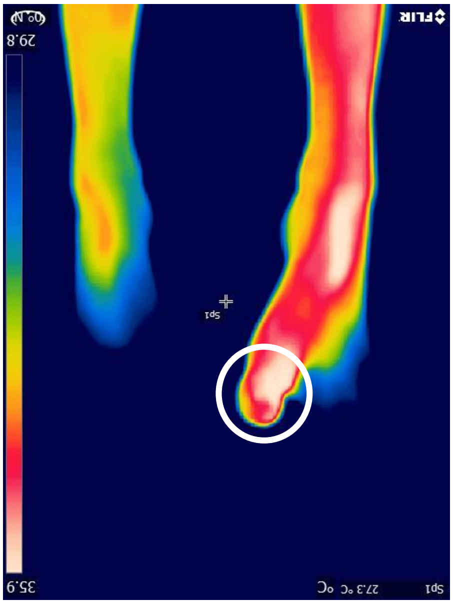

#69

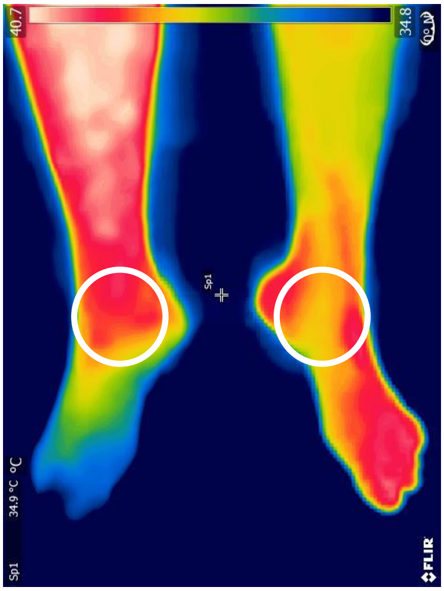


#75

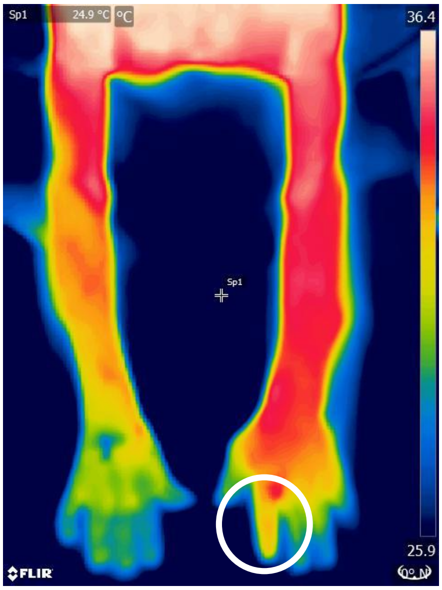


#76

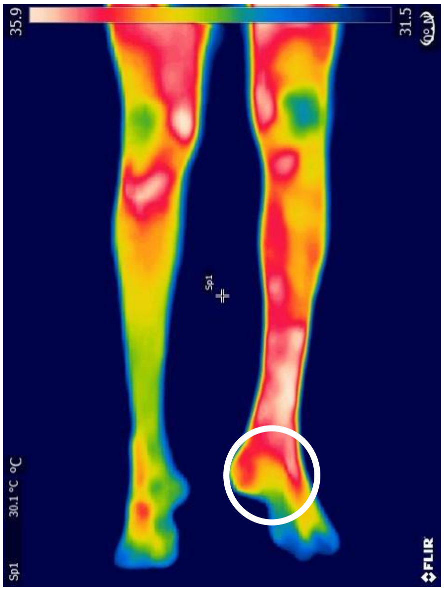


#77

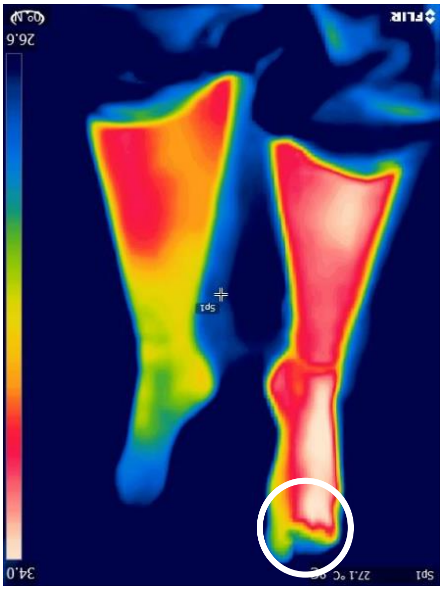

#78

# 1a. Envenomation+ Hot spot+

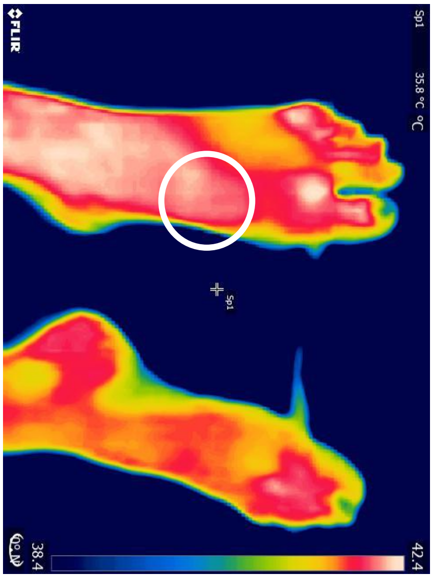

**#80**

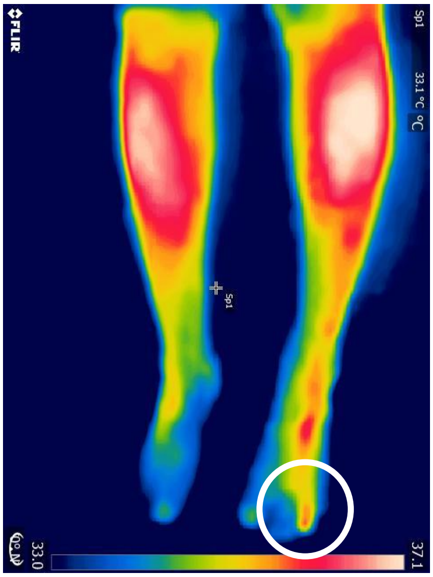

**#81**

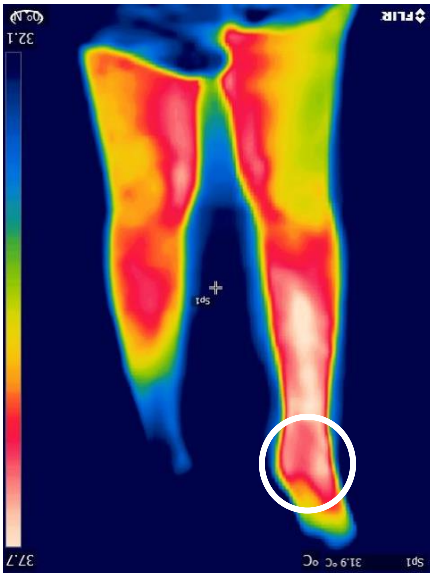

**#85**

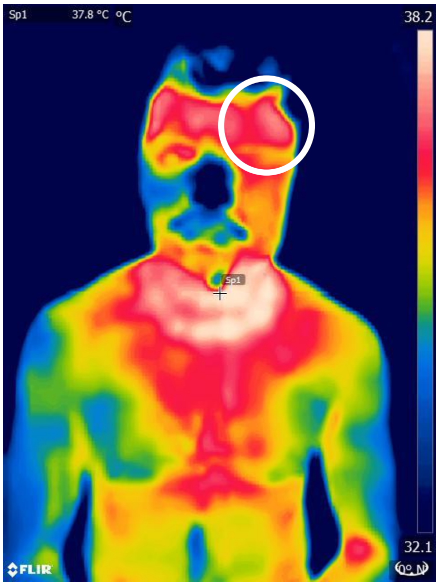


**#86**

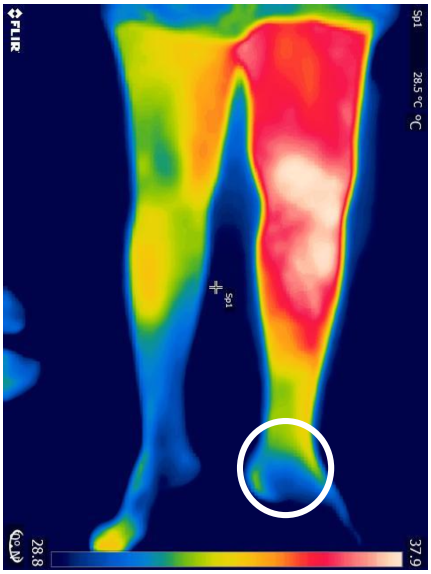

**#87**

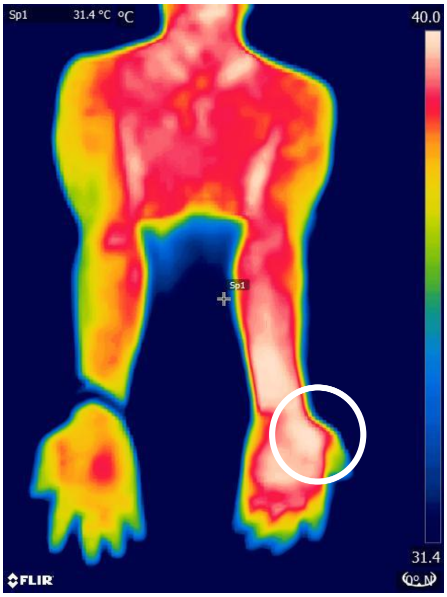


**#88**

# 1a. Envenomation+ Hot spot+

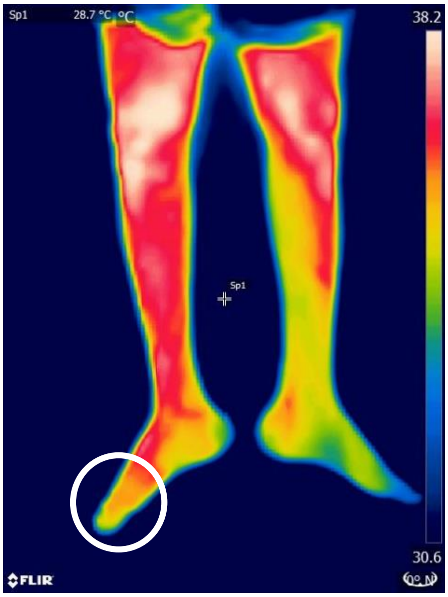


#89

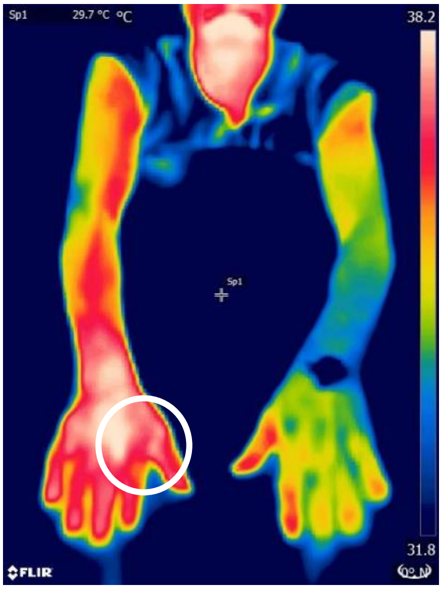


#90

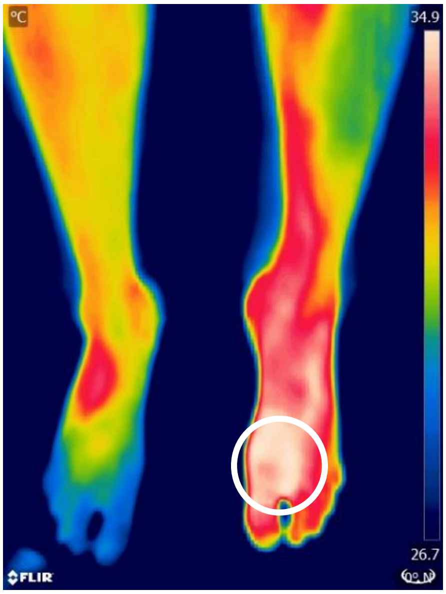


#91

# 1b. Envenomation+ Hot spot-

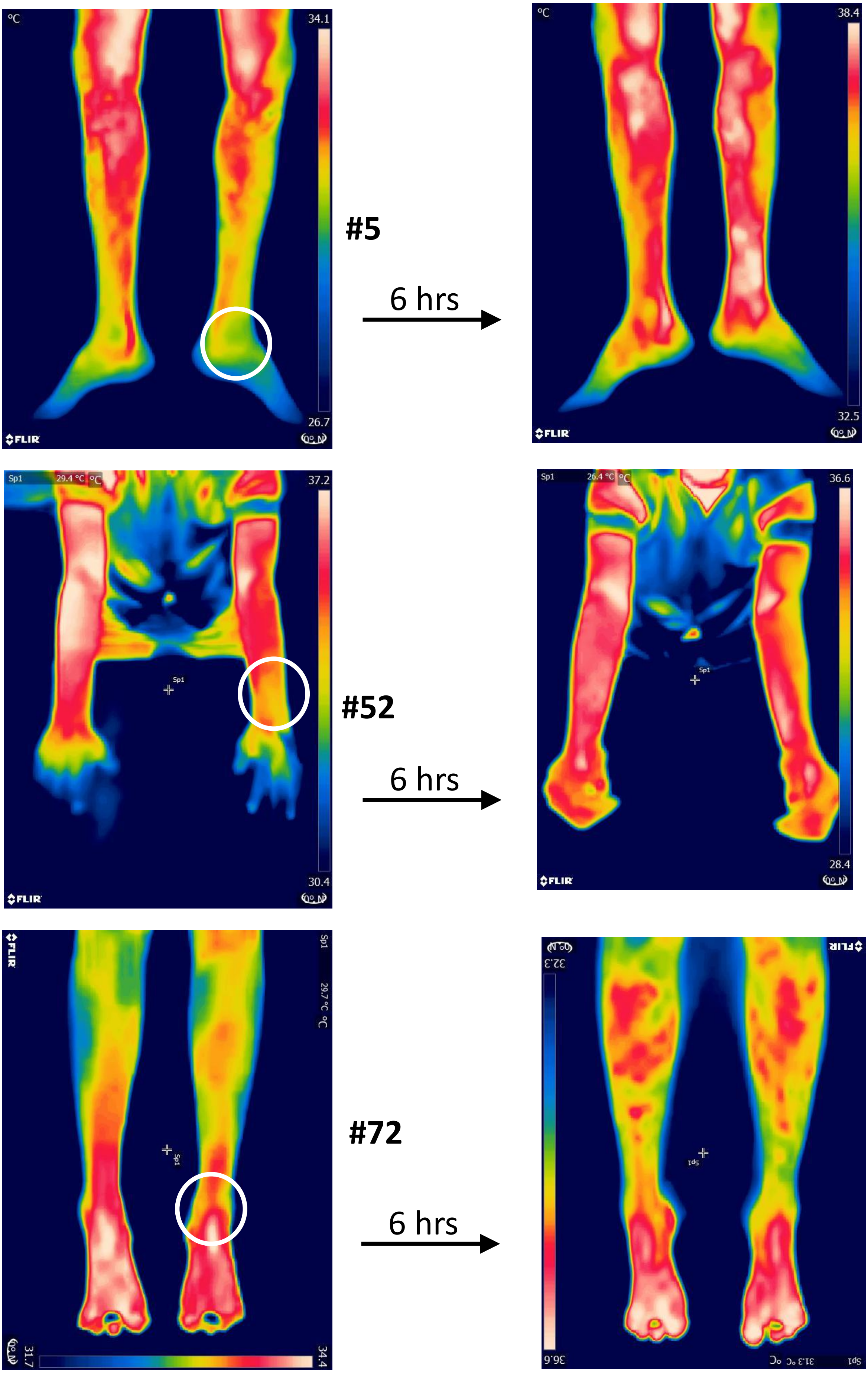

Supplement: S1 Fig — Approximate site of snakebite is indicated using white circles. Study enrolment numbers are presented alongside images. Patient #75 was bitten over both legs. (PDF) [file pntd.0008580.s001.pdf]
