# Supplementary material for: Point-of-care infrared thermal imaging for differentiating venomous snakebites from non-venomous and dry bites
Source: PLoS Negl Trop Dis. 2021 Feb 18;15(2):e0008580. doi: 10.1371/journal.pntd.0008580 (PMC7924804; doi:10.1371/journal.pntd.0008580)
Supplement: S2 Fig — Approximate site of snakebite is indicated using white circles. Study enrolment numbers are presented alongside images. (PDF) [file pntd.0008580.s002.pdf]

2a. Envenomation- Hot spot-

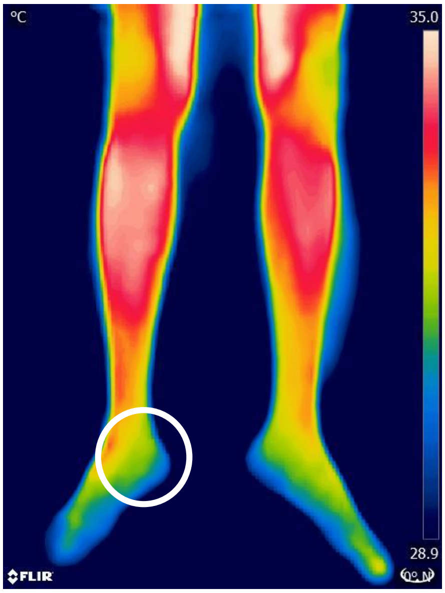

#1

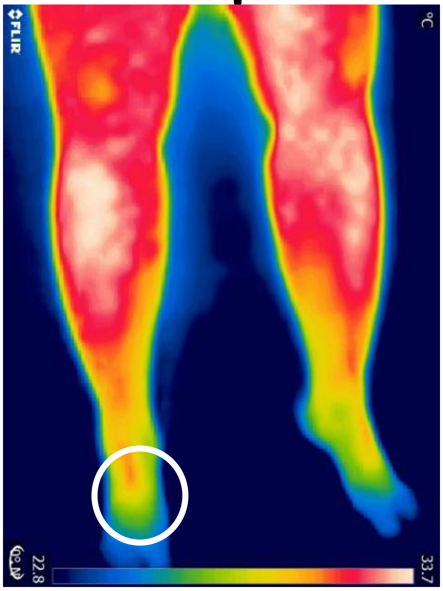

#3

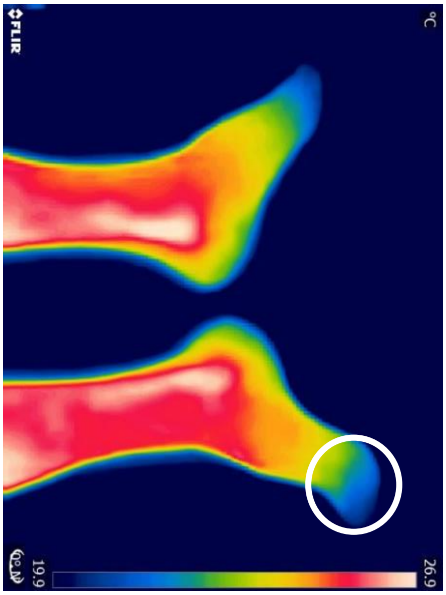

#6

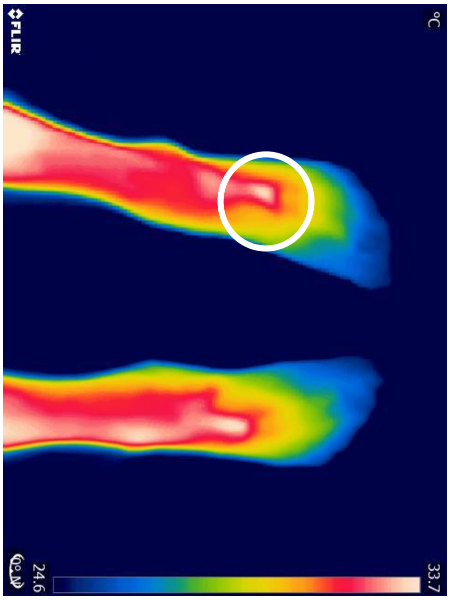

#8

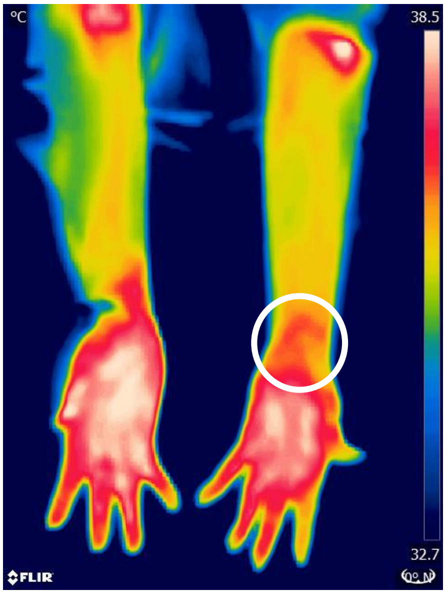

#11

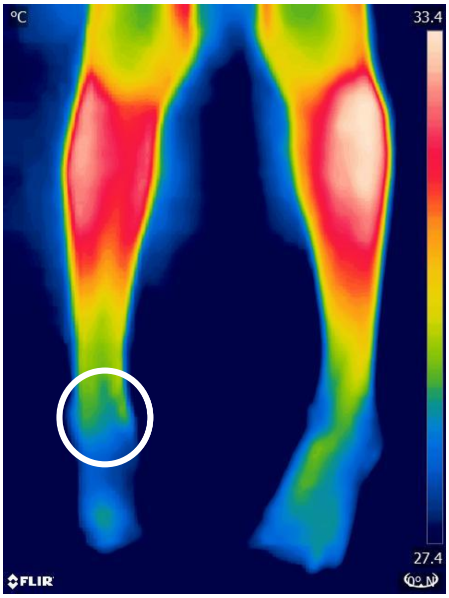

#14

2a. Envenomation- Hot spot-

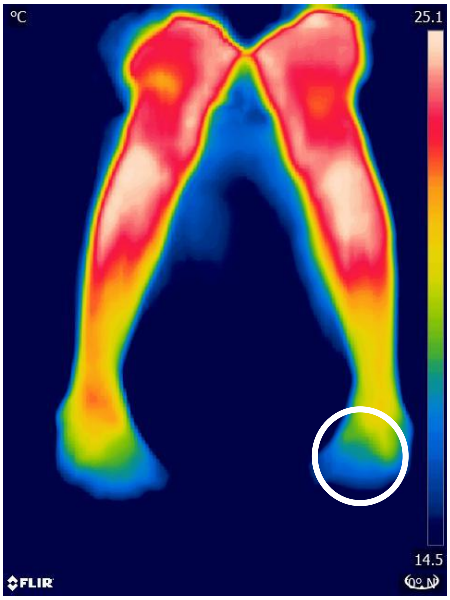

#15

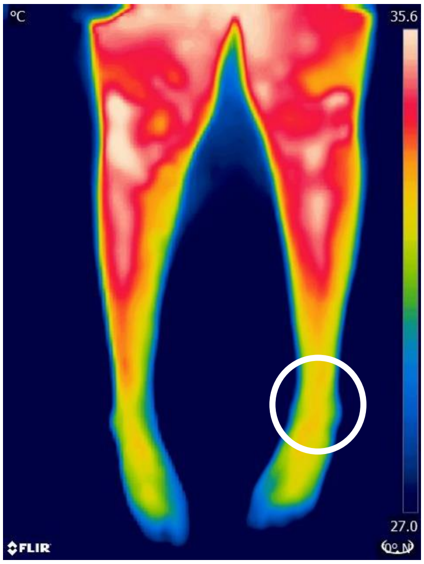

#16

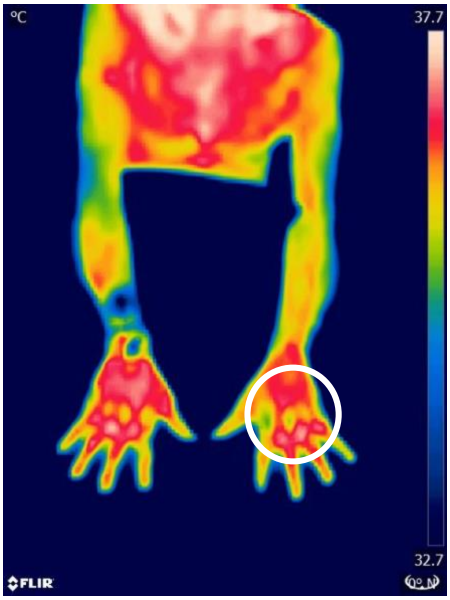

#19

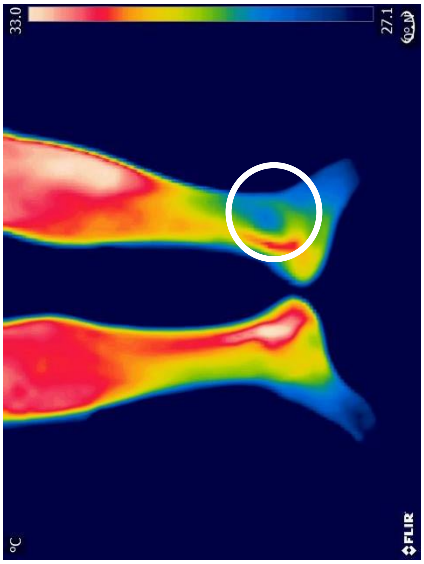

#26

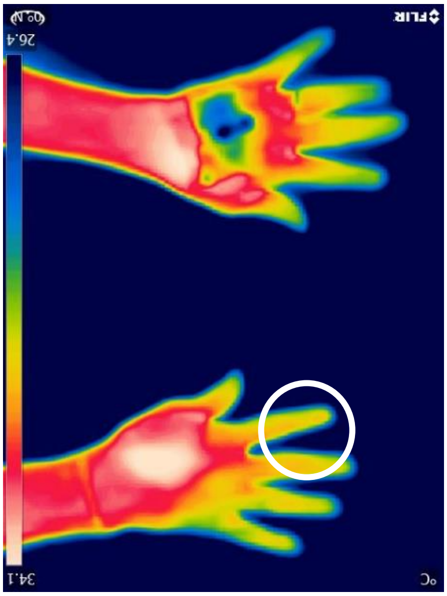

#27

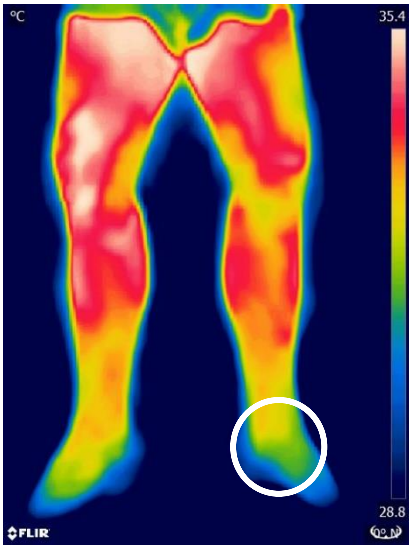

#31

2a. Envenomation- Hot spot-

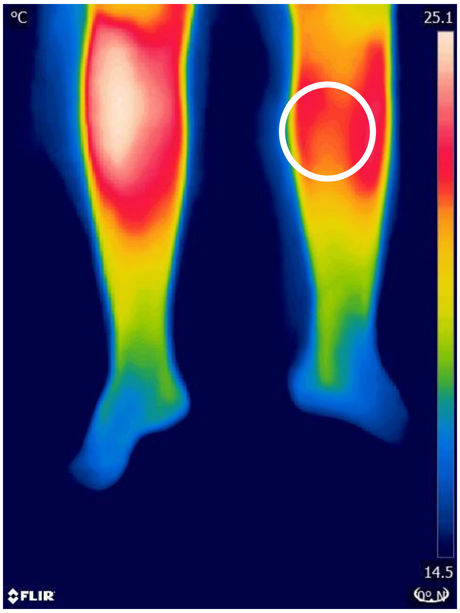

#38

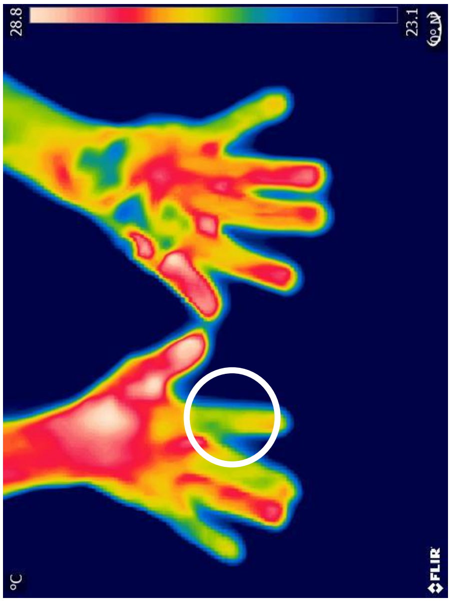

#40

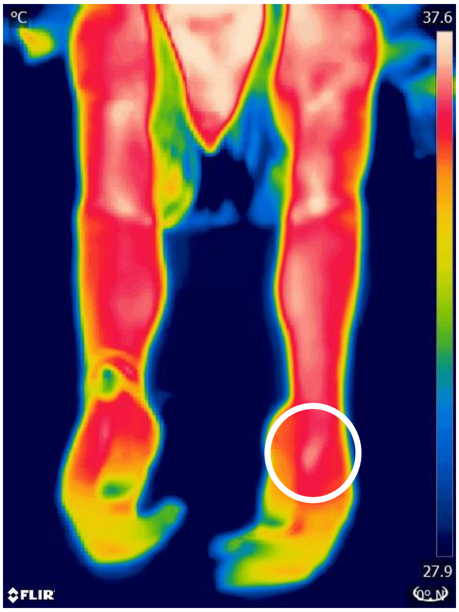

#43

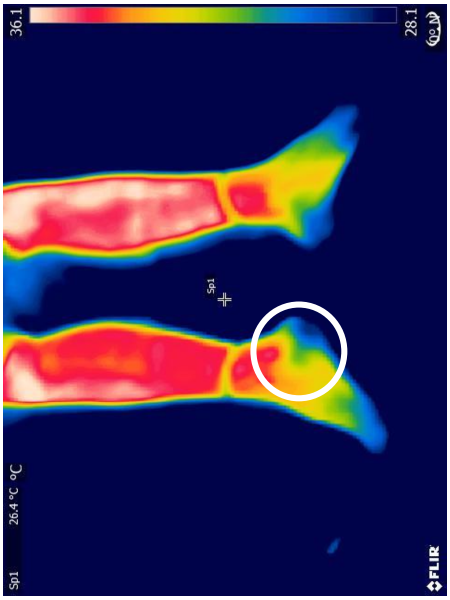

#44

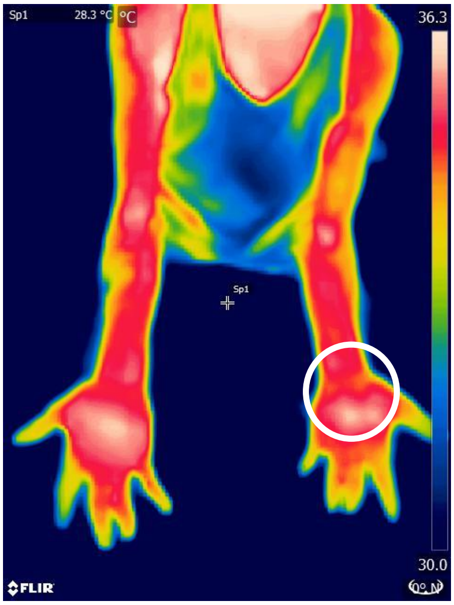

#45

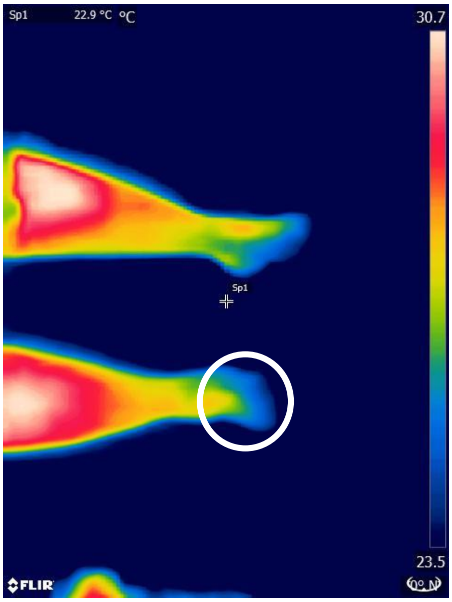

#49

2a. Envenomation- Hot spot-

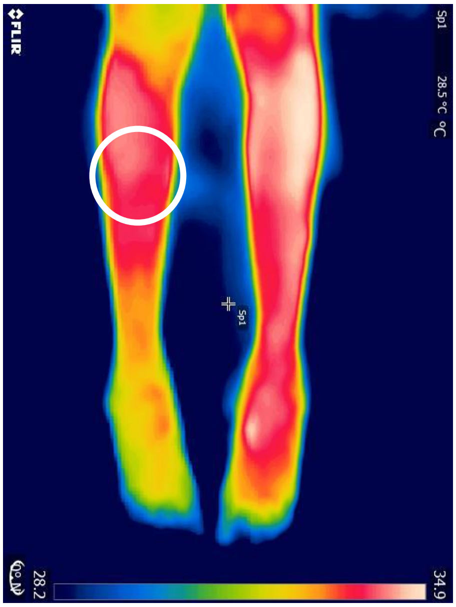

#51

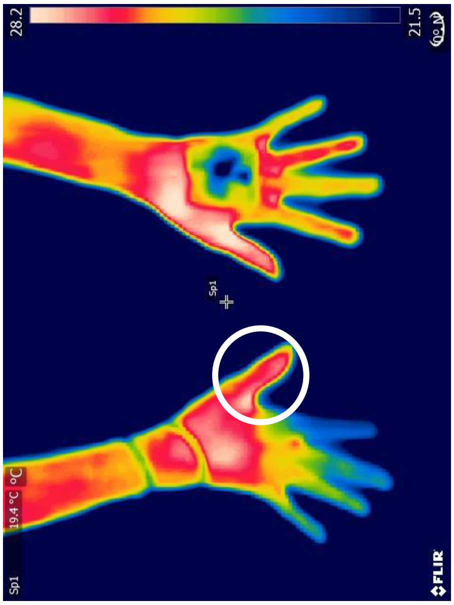

#55

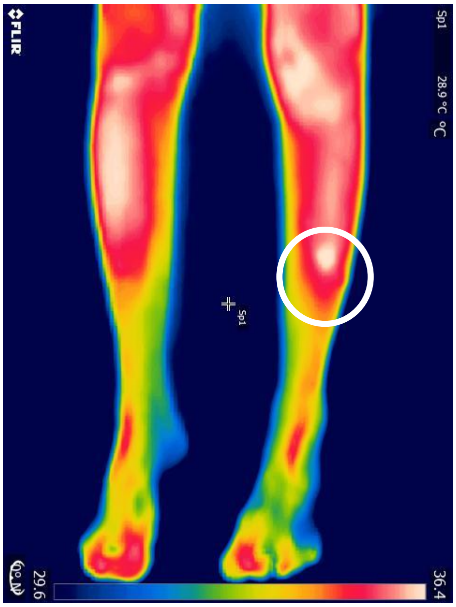

#62

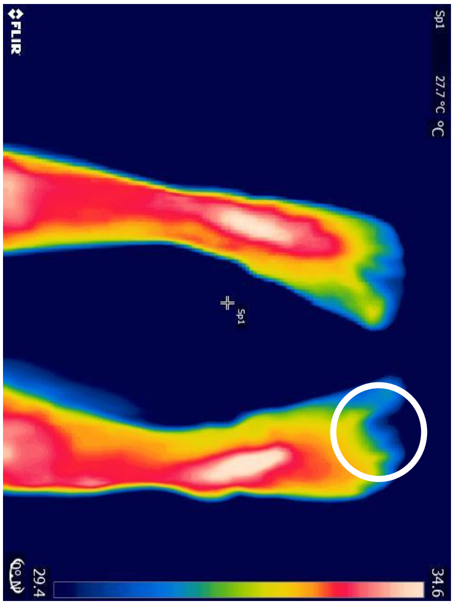

#63

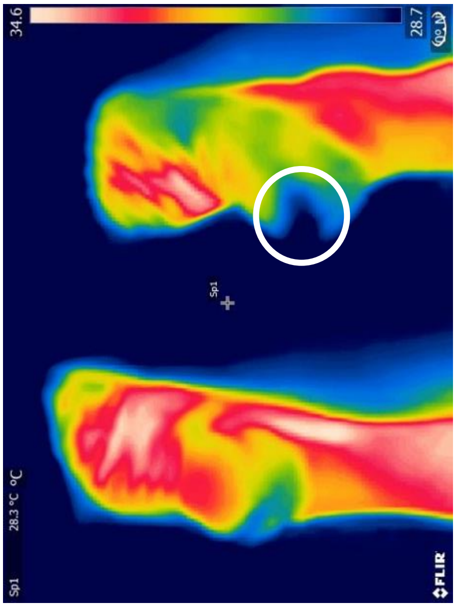

#70

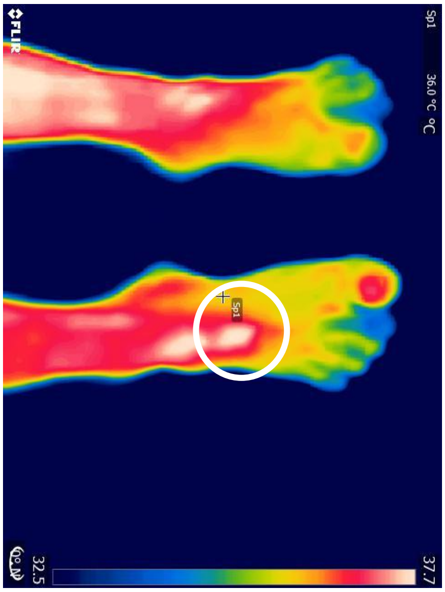

#71

2a. Envenomation- Hot spot-

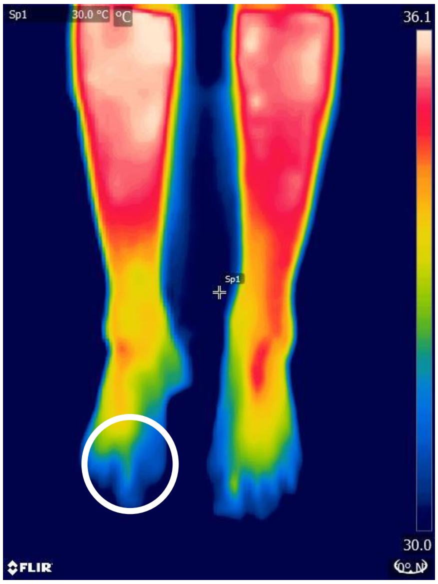

#73

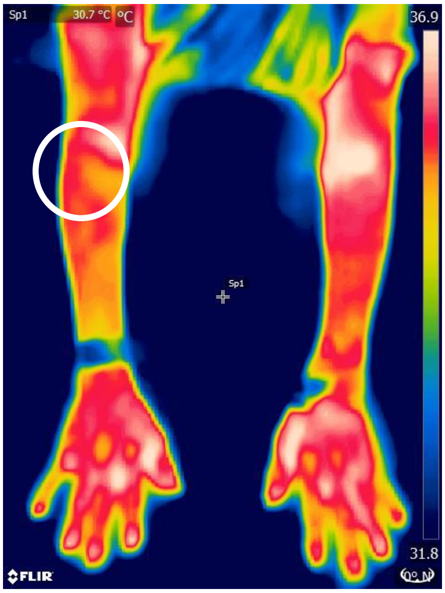

#74

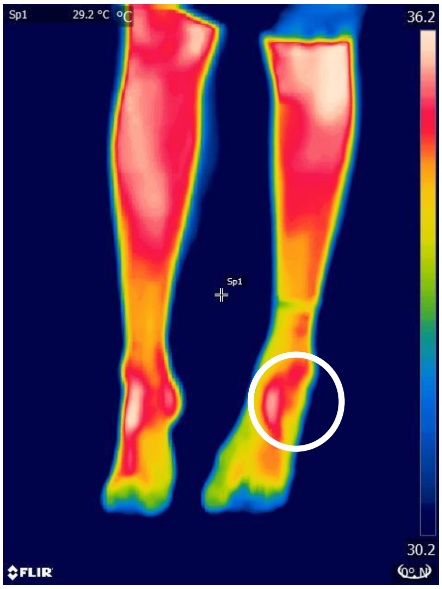

#79

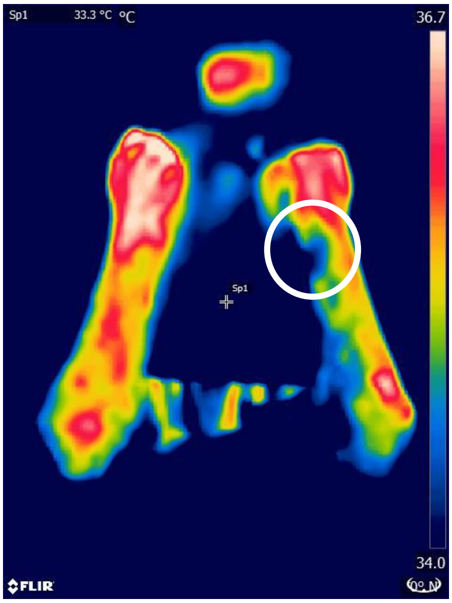

#83

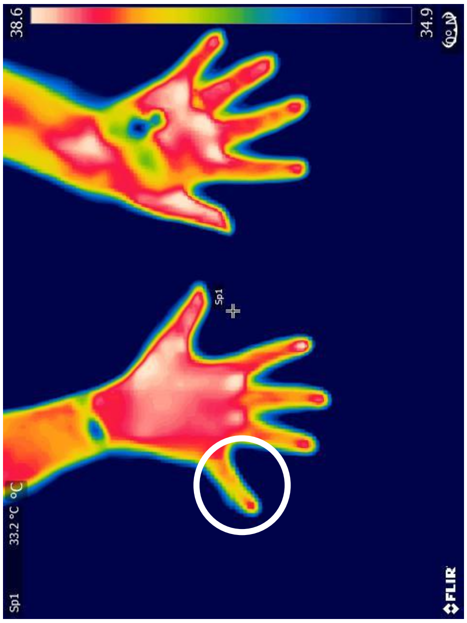

#84

2b. Envenomation- Hot spot+

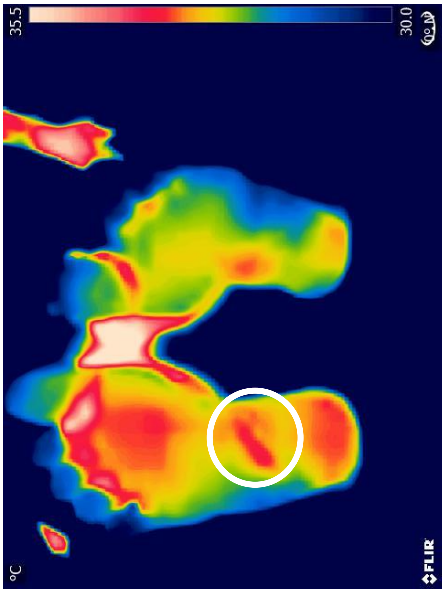

#2

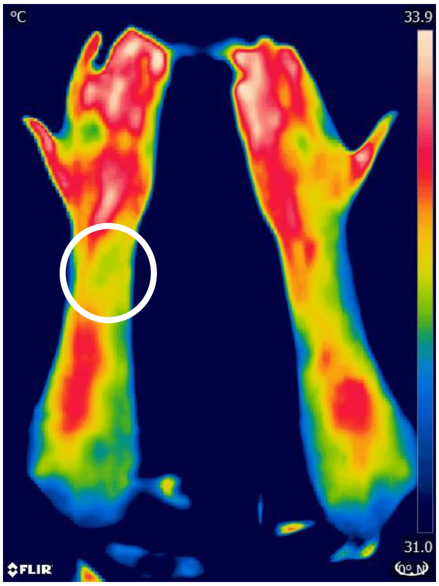

#12

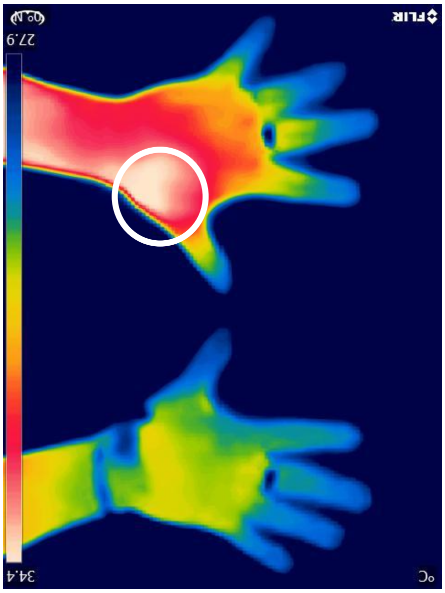

#23

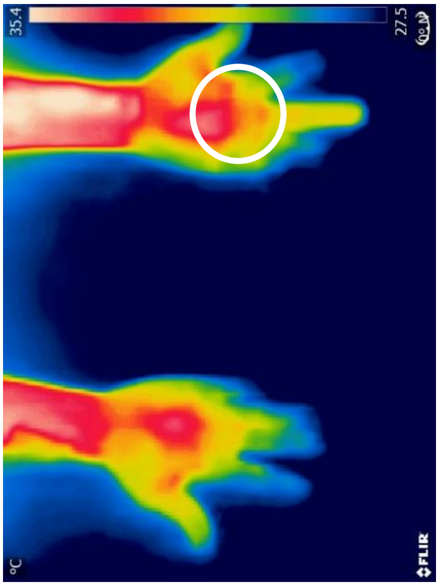

#37

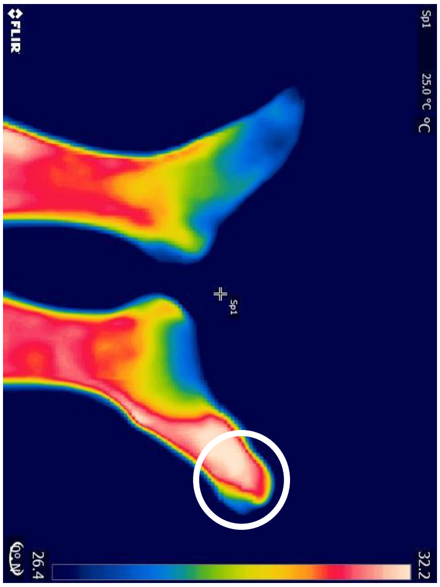

#57

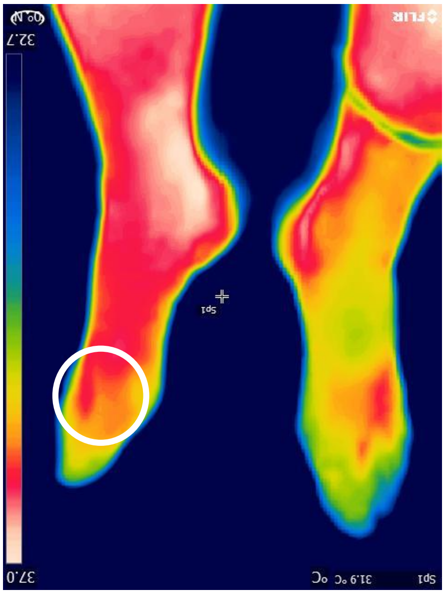

#82
