# Supplementary figures and images for: Point-of-care infrared thermal imaging for differentiating venomous snakebites from non-venomous and dry bites
Source: PLoS Negl Trop Dis. 2021 Feb 18;15(2):e0008580. doi: 10.1371/journal.pntd.0008580 (PMC7924804; doi:10.1371/journal.pntd.0008580)

# 3. Unclear envenomation status

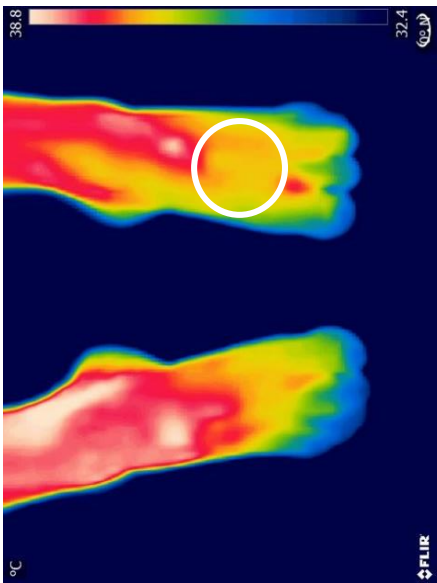

#17

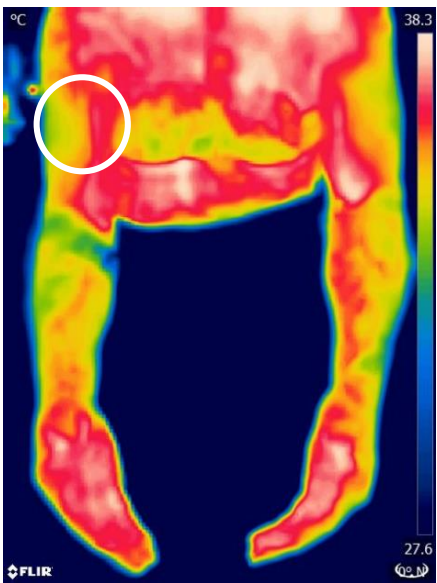

#21

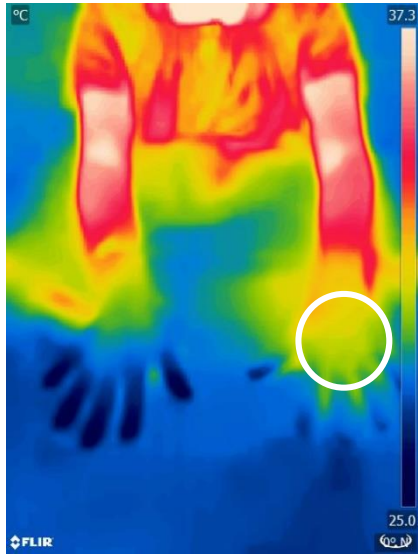

#25

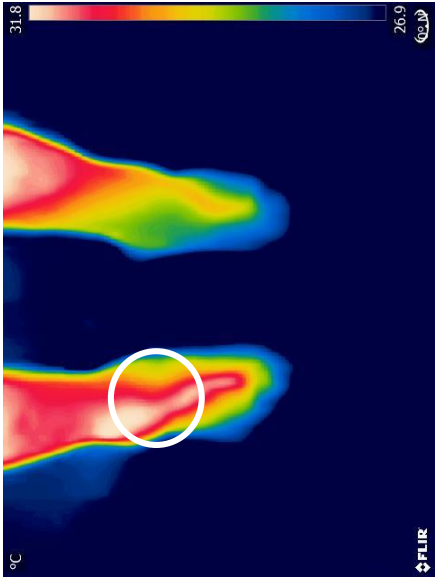

#29

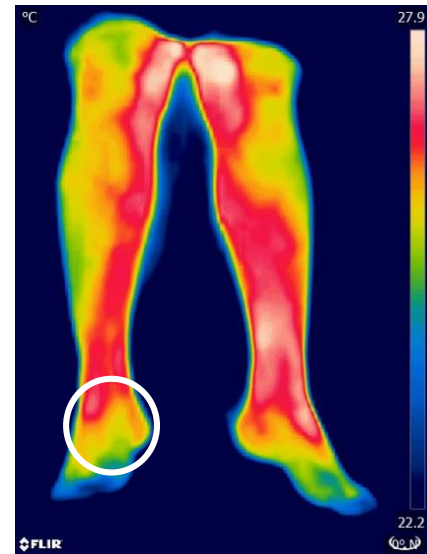

#33

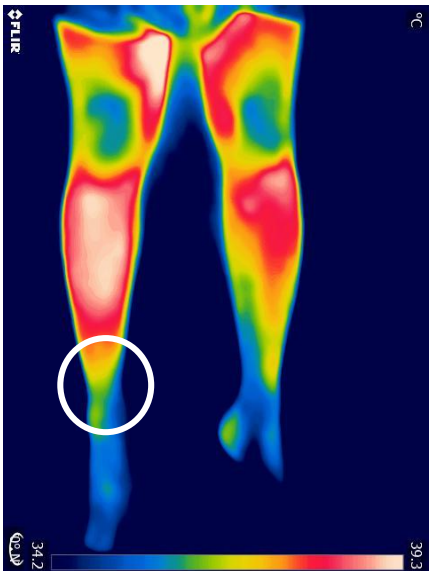

#34

Supplement: S3 Fig — Approximate site of snakebite is indicated using white circles. Study enrolment numbers are presented alongside images. (PDF) [file pntd.0008580.s003.pdf]
