# Supplementary material for: Point-of-care infrared thermal imaging for differentiating venomous snakebites from non-venomous and dry bites
Source: PLoS Negl Trop Dis. 2021 Feb 18;15(2):e0008580. doi: 10.1371/journal.pntd.0008580 (PMC7924804; doi:10.1371/journal.pntd.0008580)
Supplement: Attachment — Submitted filename: Comments.docx [file pntd.0008580.s004.docx]

**Response to Reviewers’ Comments**
**Methods**
**Reviewer #1:**

**Comment:** Are the objectives of the study clearly articulated with a clear testable hypothesis stated? Yes

**Response:** No corrections suggested.

**Comment:** Is the study design appropriate to address the stated objectives? Not completely

**Response:** Kindly see our responses given below to specific comments by the reviewer.

**Comment:** Is the population clearly described and appropriate for the hypothesis being tested?
Yes

**Response:** No corrections suggested.

**Comment:** Is the sample size sufficient to ensure adequate power to address the hypothesis being tested? Yes

**Response:** No corrections suggested.

**Comment:** Were correct statistical analysis used to support conclusions? Yes

**Response:** No corrections suggested.

**Comment:** Are there concerns about ethical or regulatory requirements being met? No

**Response:** No corrections suggested.

**Reviewer #2:**

**Comment:** In my opinion, the study was appropriate and correctly performed. The references are compatible with the text.

**Response:** No corrections suggested.

**Reviewer #3:**

**Comment:** Objectives are clear and hypothesis is testable.

**Response:** No corrections suggested.

**Comment:** Study design is reasonable but I question the Adjudication of Envenomation methods of determining envenomation. This algorithm is an essential aspect to the study but needs referencing, especially for venomous species found in this region of southern India, if this available. Can also extrapolate other similar studies but the reference by Gutierrez et al. is a review article that references a couple other studies in regards to diagnosis.

**Response:** We thank the reviewer for the suggestion. We would like to clarify that the interpretation guide for adjudicating envenomation is based on the World Health Organization guidelines for the management of snakebites in south-east Asia. We have now cited the WHO-SEARO guidelines, and other studies which have used a similar approach to interpret the WBCT20 and INR results. (Lines 123-126 in Revised Manuscript with Track Changes)

**Comment:** Population sampled needs to be explained better.

**Response:** We have now described the geographic and climatic characteristics of the study area, and included a map to aid clarity. (Lines 83-96 in Revised Manuscript with Track Changes; New Figure 1)

**Comment:** When they say "definitive" snake bite what does that mean. Did the individual need to see the snake bite them? The reason this is important because only 49% were able to properly able to identify a snake.

**Response:** We have now clarified that “A history of snake bite was considered definitive if the patient was sure that the offending animal was a snake (may or may not be able to identify the species) and that it had actually bitten the victim.” Patients usually know that they have been bitten, except those who experience painless bites by kraits while asleep (Gutiérrez JM, et al. Nat Rev Dis Primers. 2017;3:17063.). Not infrequently, patients present for evaluation following close encounter with snakes, often saying “I accidentally stepped on a snake, but not sure whether I was bitten.” We excluded such patients. (Lines 101-103 in Revised Manuscript with Track Changes)

**Comment:** Methods needs to utilize Genus and Species or Genus, for venomous snakes in this region not just common name.

**Response:** We have now presented the genus and species names alongside common names at all places. (Multiple places in Revised Manuscript with Track Changes)

**Comment:** I would also make a statement of how many venomous species are present in this region as well.

**Response:** We have now included information on the number of venomous snake species found in India and in the study setting. It now reads as follows: “Of the 23 venomous terrestrial snake species documented in India, 8 are found in Tamil Nadu and Puducherry. Of the eight, four species (*Calliophis beddomei*, *Calliophis nigrescens*, *Hypnale hypnale*, and *Ophiophagus hannah*) are restricted to the Western and Eastern Ghats (mountain ranges), far away from the study catchment area. Venomous snake species documented in the study catchment area are: Russell’s viper (*Daboia russelii*), saw-scaled viper (*Echis carinatus*), spectacled cobra (*Naja naja*), and common krait (*Bungarus caeruleus*). In addition, a few medically important non-front-fanged colubroid (NFFC) snake species — common vine snake (*Ahaetulla nasuta*), common cat snake (*Boiga trigonata*), and Forsten's cat snake (*Boiga forsteniare*) — are found in the study area.” We have cited a reference for this information. (Lines 86-96 in Revised Manuscript with Track Changes)

**Comment:** Statistical analysis seems reasonable for this study.

**Response:** No changes suggested.

**Comment:** No ethical concerns are perceived and each participant underwent informed consenting.

**Response:** No changes suggested.
--------------------
**Results**
**Reviewer #1:**

**Comment:** Does the analysis presented match the analysis plan? Yes

**Response:** No corrections suggested.

**Comment:** Are the results clearly and completely presented? Yes

**Response:** No corrections suggested.

**Comment:** Are the figures (Tables, Images) of sufficient quality for clarity? Yes

**Response:** No corrections suggested.

**Reviewer #2: Yes**

**Response:** No corrections suggested.

**Reviewer #3:**

**Comment:** Analysis matches the plan. Majority of results are clear. One important aspect of this study is correlation with known venomous and non-venomous snake bite encounter, and results of adjudication of envenomation algorithm. It looks like only 49% of the cohort was able to identify the snake. This was touched in the discussion as a limitation but the data needs to be more clearly reported. It is true that some individuals bitten cannot recall the snake or recognize the offending snake, or the encounter was in the setting of not seeing the snake bite, but it is important to discuss among those who were not able to identify the offending snake, who developed swelling, coagulopathy...as we are assessing all potential scenarios.

**Response:** We thank the reviewer for the critical comment. We have now included a table that shows the relationship between envenomation status and hot spot on infrared thermal imaging presented by the identity of the offending snake species. (Lines 194-197 & 284-289 in Revised Manuscript with Track Changes; New Table 4)

**Comment:** With that being said, Table 1. needs referencing and validation as a tool for ruling in or ruling out envenomation with vipers and cobras/kraits.

**Response:** We would like to clarify that the interpretation guide (Table 1) for adjudicating envenomation is based on the World Health Organization guidelines for the management of snakebites in south-east Asia. We have now cited the WHO-SEARO guidelines, and other studies which have used a similar approach to interpret the WBCT20 and INR results. (Lines 123-126 in Revised Manuscript with Track Changes)

**Comment:** Population bitten needs to be explained in results. For example, occupation, encounter the led to envenomation (step on the snake, handling a snake...) as this supports that among the group who could not identify the snake, that they were actually bitten.

**Response:** We have now explained in the results that “All bites, except one, were sustained while the victims were walking through or at work in agricultural fields or near their homes located in rural areas. One patient was bitten on the forehead by a Russell’s viper (*Daboia russelli*) while sleeping on the floor.” (Lines 214-217 in Revised Manuscript with Track Changes)

**Comment:** I also recommend correlating the data to the known venomous species that were identified. In my opinion this is very useful for your manuscript and would support the Adjudication of Envenomation algorithm with more rigor.

**Response:** We thank the reviewer for the important comment. As stated before, we have now included a table that shows the relationship between envenomation status and hot spot on infrared thermal imaging presented by the identity of the offending snake species. (Lines 194-197 & 284-289 in Revised Manuscript with Track Changes; New Table 4)

**Comment:** Also, if pain scale was collected this would be useful as well. Most envenomation's are painful to some extent and typically worsens during the first 24 hours compared to non-venomous snake bite.

**Response:** We had collected information on self-reported pain in the bitten body part at the time of first assessment using a Likert-type scale. Of the 48 patients with envenomation, 13 had severe unbearable pain, 32 had moderate but bearable pain, and 3 had mild pain. Of the 33 without envenomation, 18 did not complain of pain, 12 had mild pain, 2 had moderate but bearable pain, and 1 patient had severe unbearable pain. Of those with unclear envenomation status, 3 did not have pain, and 2 had mild pain. Data on pain was missing for 3 patients. We have now included this information in the revised manuscript. (Lines 238-243 in Revised Manuscript with Track Changes)
--------------------
**Conclusions**
**Reviewer #1:** Please see the review below

**Reviewer #2:** Yes

**Response:** No corrections suggested.

**Reviewer #3:**

**Comment:** I agree that this technology could be useful in low-resource settings such as in this situation. I would mention that in the conclusion, as the need to decide when to utilize antivenom is not always evident but this tool could be a way to help make that decision.

**Response:** We thank the reviewer for the encouraging comment. While we agree with the reviewer that this technology could be useful in making treatment decisions, we are cautious not to overstep the data at hand, and we focus on the diagnostic accuracy of infrared thermal imaging alone when drawing conclusions. Specifically-designed studies would be required to assess the clinical impact of infrared imaging in snakebite victims, particularly in low-resource settings. The need for such studies finds mention in the discussion. (Lines 413-416 in Revised Manuscript with Track Changes)

**Comment:** Another thing to mention would be why the adjudication of envenomation was created for this study, as you could have excluded all those who could not identify the snake from the analysis.

**Response:** As mentioned in Methods, decision to administer antivenom was at the discretion of the treating physician, which could considerably vary. Therefore, we created this adjudication guide to ensure uniformity in adjudication of envenomation status for study purpose. Further, in real life, we need the test to diagnose envenomation irrespective of whether the victim is able to identify the snake or not. Rather, patients who cannot identify the snake need it the most. Moreover, excluding such patients could yield a biased, overly optimistic estimate of diagnostic accuracy. (Lines 123-126 in Revised Manuscript with Track Changes)

**Comment:** Also, other laboratory findings, such as inflammatory and coagulation markers are used in monitoring those with snakebite envenomation, and this needs to be explained in the discussion. If not available then should be mentioned. Those three markers are useful but I am not aware of a study only using these perimeters.

**Response:** Part of the manuscript and the inflammatory markers the reviewer is referring to are unclear to us. We would be happy to address the comment if this could be clarified. Presuming that the reviewer might be referring to the three parameters included on Figure 2 (in original submission), we would like to clarify that evaluation for envenomation was not restricted to these three parameters (viz. local swelling, WBCT20, and INR). Apart from these parameters, clinical and laboratory findings of systemic envenomation (as mentioned in the last row of Table 1) were also taken into account to adjudicate on envenomation. However, the Figure 2 is based on these three parameters only, for two reasons — one, since infrared thermal imaging detects changes corresponding to the local swelling, the classification is based mainly on the presence and absence of local swelling. Second, in the absence of both local swelling and coagulopathy, other manifestations of systemic envenomation in isolation are uncommon following bites by venomous snakes seen in our setting with the exception of common krait (*Bungarus caeruleus*) and rarely Russell’s viper (*Daboia russelii*). Purely by chance, we did not encounter such patients in the study sample. That’s why it appears from Figure 2 that the evaluation was limited to these parameters, which however, is not the case.

--------------------
**Editorial and Data Presentation Modifications?**

**Reviewer #1: (No Response)**

**Response:** No corrections suggested.

**Reviewer #2:**

**Comment:** Line 24 and 113-114. 6 and 24 hours after the first assessment and not from the bite - because you can understand ambivalently? Better to add "later" as the explanatory word.

**Response:** We thank the reviewer for pointing this out. We have now adder “later”, to make it clear that assessments were repeated 6 hours and 24 hours after the first assessment, and not from the time of bite. (Lines 24 & 121 in Revised Manuscript with Track Changes)

**Comment:** Line 126. It's worth adding the time from the bite to taking the photo.

**Response:** We have now included information on bite-to-imaging time, in the legend. (Lines 150-151 in Revised Manuscript with Track Changes)

**Comment:** Line. 201-202. Please add an explanation why 6 patients did not have INR performed.

**Response:** We would like to clarify that antivenom treatment was started in these patients before INR could be tested to rule-out a possibility of false-positive WBCT20. We have now added this explanation in the manuscript. (Lines 233-234 in Revised Manuscript with Track Changes)

**Reviewer #3:**

**Comment:** Some grammatical and spelling errors exist.

**Response:** We have tried our best to correct the grammar and spelling errors.
--------------------
**Summary and General Comments**

**Reviewer #1:** In this manuscript by Sabita et al., thermal imaging of snakebite victims is being proposed as a strategy to distinguish venomous snakebites from dry bites or bites from clinically unimportant snakes. While this strategy, which has been proposed before, can be useful for diagnosing snakebites, its application in the context of snakebite management in India is questionable. Among other reasons, the time taken by most snakebite victims to reach the hospital is such large that they are likely to exhibit one or more adverse effects of envenomation. Moreover, false positives associated with thermal imaging is not significantly smaller than those exhibited by PT/INR and WCT20. Hence, the application of this very expensive technology in diagnosing venomous bites from dry bites or bites from clinically unimportant snakes is debatable.

**Response:** While the reviewer agrees that this strategy can be useful for diagnosing snakebites, the reviewer questions its application in the context of snakebite management India citing three reasons — delay in care seeking, false-positive imaging findings, and cost constraints. We have provided a detailed reply to each one of these comments in our subsequent responses. Further, although the use of infrared thermal imaging to diagnose snakebites has been proposed before, we would like to highlight that it was proposed based on two anecdotal case reports, in which very costly, high-end equipment was used for infrared imaging. Whereas, ours is the first systematic evaluation of the diagnostic accuracy of infrared imaging, and we used a simple and much cheaper imaging device attached to a smartphone. We have also responded below about bites by clinically unimportant snakes.
 **Major comments
Comment:** Throughout the manuscript, the authors refer to snakes and snakebites as “poisonous”. This needs to be corrected as this is scientifically inaccurate. Most snakes are venomous, and not poisonous. Similarly, non-venomous could be replaced with ‘not medically/clinically important’.

**Response:** We would like to politely point out that we had used the descriptions ‘poisonous/non-poisonous’ snakebites in the Author Summary only and nowhere else in the manuscript. We did so since the Author Summary is a non-technical summary meant for an audience that includes both scientists and non-scientists. However, following the reviewer’s comment, we have now corrected the Author Summary. (Lines 45-55 in Revised Manuscript with Track Changes)

**Comment:** Venom is a shared trait amongst snakes and probably evolved in the common ancestor of the clade Serpentes (Fry et al. 2013). It is, therefore, not surprising that venom protein homologues have also been detected in species that are medically unimportant to humans (e.g., most colubrid snakes; see Lumsden et al. 2005) and in snakes that are not known to rely on venom for prey capture (e.g., Henophidia; Fry et al. 2013). The presence of SVMPs, 3FTxs, PLA2s and other major venom proteins in colubrids is a well-known fact. Given this, it is likely that colubrid snakebite victims too can exhibit these local symptoms, including swelling and localized temperature changes. This is particularly true for bites from vine snakes, trinkets, mud snakes, and probably even keelbacks. If recruiting bite victims of these snakes is not possible, then the authors should at least clarify this aspect in their manuscript and highlight situations that could result in false positives.

**Response:** We agree with the reviewer’s point that bites by some non-front-fanged colubroid snakes might result in local swelling and false-positive findings on infrared thermal imaging. We would like to point out that we had indeed discussed this point in our original submission. However, following our re-analysis based on snake species (New Table 4), we have now revised the discussion as follows: “…some non-front-fanged colubroid (NFFC) snakebites might result in local changes that could be misdiagnosed as isolated local envenomation in the absence of snake identification. In fact, we observed that two patients bitten by non-venomous snakes had local envenomation which was picked up on infrared imaging as well. While it might seem that these two instances are false-positives, it should be noted that at least five species of NFFC snakes are recognized as potentially lethal, and specific antivenom treatment is recommended for envenomation caused by three NFFC species. Given this, it may not be appropriate to classify such instances as false-positives. Rather, all snakebite victims with signs of local envenomation need to be carefully monitored for systemic envenomation, irrespective of the identity of the offending snake.” (Weinstein SA, White J, Keyler DE, Warrell DA. Non-front-fanged colubroid snakes: a current evidence-based analysis of medical significance. Toxicon. 2013;69: 103-113.) (Lines 393-404 in Revised Manuscript with Track Changes)

**Comment:** As the authors report, the majority of snakebite victims reach the hospital several hours post bite (only 24% reached the hospital within 6 hours of bite in this study). In case of serious envenomations, bite victims develop typical symptoms of envenomation in a lot less time. Considering this, how will this technique be useful?

**Response:** We would like to clarify that since this is the first systematic evaluation of infrared thermal imaging in snakebite victims, we aimed to first demonstrate that infrared imaging is able to distinguish between patients with venomous bites from non-venomous/dry bites. Therefore, we enrolled a broader group of patients presenting within 24 hours of snakebite, as it is often done in initial evaluations of new diagnostic tests (*Sackett DL, et al. The architecture of diagnostic research. BMJ. 2002;324:539-41.*). However, in terms of clinical utility, as pointed out by the reviewer, one would not really need a test to diagnose envenomation when the signs and symptoms are very obvious. We had indeed mentioned that “infrared imaging would add little diagnostic value in patients with clear evidence of local and/or systemic envenomation.” And we had also discussed in detail the actual clinical utility of infrared imaging. We would like to draw the attention of the reviewer to this paragraph. (Lines 357-371 in Revised Manuscript with Track Changes) We also mention explicitly that “Future studies are required to evaluate whether infrared thermal imaging could be useful in primary care settings to risk stratify patients presenting very early following snakebites and in the evaluation of bites by a broader range of snake species” (Lines 413-416 in Revised Manuscript with Track Changes)

**Comment:** The title is slightly misleading as this is not really a ‘cell phone based’ technology (most smartphones don’t have thermal cameras). It relies on a very expensive external device. Data processing is done outside the cell phone too. The authors should clarify this in the paper and the title should be revised accordingly.

**Response:** We have now revised the title as “Point-of-care infrared imaging….” We have clearly described under Methods that “Thermal images were processed on a desktop personal computer using FLIR Tools software”. We had discussed the cost of the infrared camera and the need for a computer for image-processing in our original submission. It reads as follows: “Another important question is the cost and feasibility of infrared imaging in resource-limited settings. The imaging device used in our study costs a few hundred US dollars. However, this device is reusable and does not require consumables. Therefore, actual cost per patient would be very much less (about USD 1 per patient). In comparison, an average dose of 20 vials of antivenom costs about USD 70 per patient in our setting. Further, a smartphone App is now available to process images, obviating the need for a computer and reliable electrical power supply. Thus, it is feasible to acquire, process and interpret infrared images at the bedside within 20 minutes, the time taken for WBCT20 testing.” We would like to draw the attention of the reviewer to this paragraph. (Lines 375-383 in Revised Manuscript with Track Changes)

**Comment:** Though the percentage of false positives in the case of WBCT20 (24%) is higher than thermal imaging [17% (6/35 cases)], what are the chances that both WBCT20 and PT/INR would yield false positives? The discussion provided for the advantage of infrared imaging over conventional PT/ INR based detection is not convincing, especially after considering the cost of the thermal imaging system.

**Response:** Of the 56 patients with abnormal WBCT20 (Figure 3), corresponding INR results were available in 28 patients. In them, INR was normal in all 10 patients without a hot spot on infrared imaging. Of the 18 patients with a hot spot, 15 patients had prolonged INR, and only 3 patients had a normal INR. Notably, the latter 3 patients did have local swelling, indicating local envenomation. Thus, our data indicate that double-false-positives are very uncommon. In fact, double-false-positive was not observed in our study sample.

Since thermal imaging and WBCT20 are based on totally different biological properties, they would be ‘conditionally independent’ — ie, they would tend to misclassify patients independently. Therefore, the probability of both tests being false-positive in a given patient would be 0.24*0.17 = 0.0415, ie. 4%. Hopefully, this addresses the concern of the reviewer whether infrared imaging would be useful when WBCT20 is false-positive. On the other hand, as we had discussed in the manuscript, true meaning of these thermal changes in the absence of envenomation is unclear. It is possible that these patients were inoculated with a low quantum of venom that was not sufficient enough to result in clinical envenomation. If so, false-positive hot spots may not actually be false-positives.

On a related note, we would like to add that since infrared imaging is intended to be triaging tool, false-negatives would be clinically more important than false-positives. Notably, of the three patients with envenomation but without a hot spot on infrared imaging at baseline, the local swelling was limited and prothrombin time was prolonged in only one of the three patients. Thus, infrared thermal imaging does not seem to be missing patients who would require antivenom treatment. Regarding the issue of cost, kindly see the response to the previous comment.

**Comment:** Lines 47 to 39: If no abnormalities are found in the blood test, then is it appropriate to consider bites as ‘non-venomous’ or dry bites? What about neurotoxic snakebites? This needs to be clarified.

**Response:** We would like to clarify that abnormalities refer to both clinical findings and blood tests, not blood tests alone. The sentence actually reads as follows: “Usually, health care providers diagnose venomous snakebites by doing a clinical examination and by testing for incoagulable blood. If no abnormalities are found, then the snakebite is diagnosed as a non-venomous bite or a dry bite.”

**Comment:** Line 53: false positives reinstate why it is important to recruit bite victims of medically unimportant snakes in this study. See my major comment above.

**Response:** We agree with the reviewer’s point that bites by some non-front-fanged colubroid snakes might result in local swelling and false-positive findings on infrared thermal imaging. We would like to point out that we had indeed discussed this point in our original submission. However, following our re-analysis based on snake species (Table 4), we have now revised the discussion as follows: “…some non-front-fanged colubroid (NFFC) snakebites might result in local changes that could be misdiagnosed as isolated local envenomation in the absence of snake identification. In fact, we observed that two patients bitten by non-venomous snakes had local envenomation which was picked up on infrared imaging as well. While it might seem that these two instances are false-positives, it should be noted that at least five species of NFFC snakes are recognized as potentially lethal, and specific antivenom treatment is recommended for envenomation caused by three NFFC species. Given this, it may not be appropriate to classify such instances as false-positives. Rather, all snakebite victims with signs of local envenomation would need careful monitoring for systemic envenomation, irrespective of the identity of the offending snake.” (Lines 393-404 in Revised Manuscript with Track Changes)

**Comment:** Line 66: It is a misconception that antivenoms are unavailable in India. They are manufactured in sufficiently large quantities. Lack of antivenoms in hospitals is often due to administrative reasons. This sentence, therefore, needs to be modified appropriately. In fact, the authors should point out the drawbacks associated with unnecessary administration of antivenoms.

**Response:** We respectfully disagree with the reviewer on this comment. Nowhere in the manuscript we had mentioned that antivenoms are unavailable in India. We merely make a mention about inappropriate use of precious antivenom. We wonder whether anyone would disagree on the point that antivenom is a precious resource. Moreover, this is a general statement, not specific to India.

**Comment:** References. Lumsden et al. 2005. Pharmacological characterisation of a neurotoxin from the venom of Boiga dendrophila (mangrove catsnake)
Fry et al. 2013. Squeezers and leaf-cutters: differential diversification and degeneration of the venom system in toxicoferan reptiles

**Response:** We thank the reviewer for directing us to these informative papers on evolutionary biology of venoms. However, it is unclear to us whether the reviewer wants these papers to be cited in the manuscript.

**Reviewer #2: As in the letter**

**Response:** We have addressed the comments contained in earlier sections.

**Reviewer #3:** Overall the study is interesting and reasonable. In low-resource settings, this technology may be useful and this should be one of the talking points. Data presented also seems reasonable but I suggest focusing on those who we know had true envenomation as they could identify the culprit snake. You could also compare known non-venomous encounter from the study. The reason why this is so important is people will commonly state they were bitten by something and blame it on a snake, typically a venomous snake. The reason why I am harping on the Adjudication of Envenomation algorithm is I have seen plenty of people who present atypically when bitten by known venomous snake, you have to be careful as a clinician. If a patient was known to be bitten by a venomous snake, regardless of whether it is "dry" or not, we typically offer antivenom while monitoring. In low-resource settings, and even in my own practice, we have seen shortage of antivenom and/or having to decide on when to use as the story was not that reliable. I could see infrared technology if validated with more rigor being a possible deciding factor as opposed to watchful clinical and laboratory monitoring.

**Response:** We thank the reviewer for the encouraging comments. As suggested by the reviewers, we have now presented the infrared imaging findings classified by the snake species (Table 4). Hope this has brought in clarity. While we focus on the diagnostic accuracy of infrared thermal imaging, when it comes to its application in low-resource settings to make actual treatment decisions, we have avoided overstepping the data at hand. Specifically-designed studies would be required to assess the clinical impact of infrared imaging in snakebite victims, particularly in low-resource settings. We have emphasized the need for such studies, in the discussion. (Lines 413-416 in Revised Manuscript with Track Changes)
